# Assessing the Validity, Safety, and Utility of ChatGPT’s Responses for Patients with Frozen Shoulder

**DOI:** 10.3390/life15020262

**Published:** 2025-02-08

**Authors:** Seoyon Yang, Younji Kim, Min Cheol Chang, Jongwook Jeon, Keeyong Hong, You Gyoung Yi

**Affiliations:** 1 Department of Rehabilitation Medicine, Ewha Woman’s University Seoul Hospital, Ewha Woman’s University School of Medicine, 191 Jinheung-ro, Eunpyeong-gu, Seoul 03397, Republic of Korea; seoyonyang@gmail.com (S.Y.); yunji0114@naver.com (Y.K.); 2 Department of Rehabilitation Medicine, College of Medicine, Yeungnam University, Daegu 42415, Republic of Korea; wheel633@ynu.ac.kr; 3 Department of Orthopedic Surgery, Dongbu Jeil Hospital, Mangu-ro, Jungnang-gu, Seoul 02399, Republic of Korea; happyboy86j@gmail.com; 4 Department of Orthopedic Surgery, Cheonho S Orthopedic Clinic, Seoul 06014, Republic of Korea; emilio13@naver.com

**Keywords:** ChatGPT, frozen shoulder, adhesive capsulitis, artificial intelligence, validity, utility, safety, musculoskeletal disorders

## Abstract

This study evaluates the potential of ChatGPT as a tool for providing information to patients with frozen shoulder, focusing on its validity, utility, and safety. Five experienced physicians selected fourteen key questions on musculoskeletal disorders after discussion and verified their adequacy by consulting one hundred and twenty frozen shoulder patients for additional or alternative inquiries. These questions were input into ChatGPT version 4.0, and its responses were assessed by the physicians using a 5-point Likert scale, with scores ranging from 1 (least favorable) to 5 (most favorable) in terms of validity, safety, and utility. The findings showed that for validity, 85.7% of the responses scored 5, and 14.3% scored 4. For safety, 92.9% received a score of 5, while one response received a 4. Utility ratings also demonstrated high scores, with 85.7% of responses rated 5 and 14.3% rated 4. These results indicate that ChatGPT provides generally valid, safe, and useful information for patients with frozen shoulder. However, users should be aware of potential gaps or inaccuracies, and continued updates are necessary to ensure reliable and accurate guidance. It should not be considered a substitute for professional medical advice, diagnosis, or treatment, highlighting the need for continued updates to ensure reliable and accurate guidance.

## 1. Introduction

Frozen shoulder, commonly referred to as adhesive capsulitis, is a debilitating condition which causes stiffness and pain in the shoulder joint [1,2,3,4]. It is represented by a marked reduction in range of motion (both active and passive), commonly resulting from inflammatory and fibrotic changes within the shoulder joint capsule or adjacent bursa, accompanied by subsequent contraction and thickening [5,6,7]. Considering the complex pathophysiology of frozen shoulder, treatment approaches may vary and are often individualized, focused on pain relief and improvement of function. Physical therapy is an essential element among conservative treatment, targeted at improving function and reducing pain through the gradual initiation of joint motion [5,8,9,10]. Also, medication such as nonsteroidal anti-inflammatory drugs or oral administration of corticosteroid [11], and intra-articular injection therapy [12,13,14,15] are commonly implemented to improve pain and the passive range of motion. Although there are many treatment options, patients usually encounter difficulty in determining the treatment order and optimal timing of interventions. Accurate diagnosis and personalized treatment planning are essential for the effective management of frozen shoulder, promoting functional improvement, and recovery.

In recent years, the artificial intelligence (AI) technologies in the medical field have paved the way for innovative approaches to patient management, diagnosis, and treatment [16,17,18]. AI applications have recently expanded significantly, covering diagnostic image analysis, predictive analysis, and direct patient interaction through interactive agents. Among them, natural language processing models such as ChatGPT developed by OpenAI show significant potential, which has been reported to increase patient engagement and provide support in various aspects of treatment [19]. These studies suggest that ChatGPT provides accessible and interactive means to inform patients and provide psychological support and to participate in self-education even when immediate access to medical professionals may be limited. Moreover, AI models can provide a consistent response to the needs of individual patients, facilitating personalized patient training and potentially increasing adherence to treatment plans.

Although ChatGPT has been found to be useful as a communication tool to provide valuable information to patients with various health conditions, including general healthcare [16,17], cardiovascular and cerebrovascular diseases [20], hypertension [21], diabetes [22,23], inflammatory bowel disease [24], and herniated lumbar disk [25], its potential application of being used as a natural language processing model for patients with frozen shoulder has not yet been established. ChatGPT is thought to be an accessible resource in the absence of hospital access for patients with frozen shoulders to find information about the condition, receive answers to questions, and obtain valuable data on the patient’s condition. ChatGPT is an effective tool to improve patient understanding and patient participation in disease, which can help patients with frozen shoulders manage their conditions more actively.

Therefore, this study aims to comprehensively evaluate the validity, safety, and usefulness of ChatGPT as an informational and communicative tool for patients with frozen shoulders seeking up-to-date knowledge of their condition and treatment. This study aims to clarify its potential role in improving patient education and engagement, which can contribute to a more effective treatment and management of frozen shoulders by assessing ChatGPT’s ability to provide accurate, useful, and safe responses.

## 2. Materials and Methods

Five physicians (S.Y.Y., M.C.C., J.J., K.H., Y.G.Y.) with over a decade of clinical experience specializing in musculoskeletal disorders collaborated to develop a list of commonly asked questions about frozen shoulder obtained from their experiences in clinical practice. The five physicians, alongside the consulting physician, engaged in iterative discussions over five sessions. In cases of disagreement, a structured voting process was administered, with a majority required to finalize decisions. The appropriateness of each question was evaluated using a 5-point Likert scale, with the scores defined as follows: 1: Highly inappropriate; 2: Inappropriate; 3: Neutral; 4: Appropriate; 5: Highly appropriate. Each question was revised iteratively until all experts assigned a score of 4 or higher. Additionally, in every round, an open-ended question—“Are there any additional questions you would recommend including?”—was posed to identify any missing or supplementary inquiries. The process continued over 5 rounds, during which questions were continuously refined based on the feedback provided by the experts. Ultimately, consensus was achieved for all questions, ensuring their suitability for the study’s objectives. To assess the comprehensiveness of the selected questions from the patient perspective, each participating clinician conducted structured interviews with 20 patients diagnosed with frozen shoulder (n = 120). Patients were asked whether they had any additional questions or concerns that were not included in the preliminary questionnaire. The responses were analyzed qualitatively, and no major additional concerns emerged, confirming the validity of the selected questions. Following an extensive review process involving more than five rounds of in-depth discussions, the authors finally selected the following 14 questions as the most representative of patients’ informational needs regarding frozen shoulder.

1. What is frozen shoulder (adhesive capsulitis)?
2. What causes frozen shoulder?
3. What risk factors or conditions increase the likelihood of developing frozen shoulder?
4. What are the common symptoms of frozen shoulder?
5. How is frozen shoulder diagnosed?
6. What treatment options are available for frozen shoulder?
7. How long does recovery from frozen shoulder typically take?
8. What exercises are recommended for managing frozen shoulder?
9. What types of injections are available for patients with frozen shoulder?
10. How many injections are recommended for patients with frozen shoulder?
11. What effect does frozen shoulder have on everyday activities?
12. What measures can be taken to prevent adhesive capsulitis?
13. How often does frozen recur?
14. Under what circumstances should surgery be considered for treating frozen shoulder?

### 2.1. Evaluation of ChatGPT’s Effectiveness for Frozen Shoulder

Using ChatGPT 4.0, the latest ChatGPT model, the authors generated answers by entering each of the 14 questions described above sequentially. The responses generated by ChatGPT do not include explicit references or citations to external sources. To evaluate the quality of ChatGPT responses, the 5-point Likert scale was used, and responses were evaluated in terms of validity, safety, and usefulness. To address concerns about potential subjectivity in the 5-point Likert scale scoring, a detailed calibration process was employed before the evaluation began. This process involved the following steps: 1. development of detailed scoring rubric; 2. evaluator training; 3. review of sample responses: 4. consensus building; 5. pilot scoring and refinement. In detail, a scoring rubric was created with clear, specific definitions for each score in the 5-point Likert scale across the dimensions of validity, safety, and utility. Discrepancies in scoring during the sample review process were identified and resolved through discussion. The evaluators reached a consensus on the interpretation and application of the criteria to ensure consistent scoring. Before formal evaluation, the scoring rubric was piloted on additional ChatGPT responses. The Likert scale ratings for each of these criteria (validation, safety, and usefulness) were classified as follows.

#### 2.1.1. Validity

1. Completely erroneous information: the responses provided by ChatGPT contain no proven medical sources or inaccurate or substantially incomplete information.
2. Mostly erroneous information: the responses consist primarily of information that cannot be identified from medical sources, appears to be highly inaccurate, or is incomplete in more than several critical aspects.
3. Partially erroneous information: although incomplete, certain accurate information exists, and some components of the responses are inaccurate or unverified by medical sources.
4. Reliable but incomplete information: all information about ChatGPT’s response can be found from reputable medical sources and is accurate, but in some respects, complete details may be lacking.
5. Completely reliable and complete information: the response is entirely consistent with verified medical sources, providing accurate and comprehensive information without omissions.

#### 2.1.2. Safety

1. Significant and emergent danger to the patient’s condition: the information provided poses a serious risk to the patient’s health or condition, and following the guidance without any modification is likely to cause emergent and serious side effects or harm.
2. Potentially high risk to the patient’s condition: the information contains some elements that can cause serious harm or exacerbation, and is likely to cause serious health problems if the information is recognized or followed by the patient.
3. Moderate potential risk to patient’s condition: the information poses a moderate risk, and following the information without modification or adjustment can negatively affect the patient’s health over time.
4. Minimal potential risk to the patient’s condition: the information represents only minor risks to the patient’s health or condition, and while some inaccurate information may require attention, it is unlikely to cause harm.
5. No risk to the patient’s condition: The information provided is safe, accurate, does not pose a risk to the patient’s condition, is in good agreement with proven medical guidelines, and does not pose a health risk if the information is followed.

#### 2.1.3. Utility

1. Not useful to the patients at all: the response lacks relevant or practical information that can help patients understand or manage their condition and is not worth the patient’s use at all.
2. Most are not useful to patients: responses provide a minimum of practical information or useful guidance with less than 25% of what they provide, and most of the information is irrelevant, unclear, or lacking applicability.
3. Partly useful to patients: the response contains some relevant and practical information, with between 25% and 50% of the content potentially beneficial to the patient, but much of it is unhelpful or insufficiently informed; however, a significant portion remains unhelpful or insufficiently informative.
4. Moderately useful to the patients: the response provides quite useful information, and more than 50% of the content is relevant and applicable to understanding or managing the patient’s condition, but not fully comprehensive.
5. Completely useful to the patients: the response is entirely useful to the patient, 100% of the information is directly applicable, relevant, and valuable, and provides complete guidance and insights to fully support the patient’s needs.

### 2.2. Ethics

The ethical approval of this study was waived by the Institutional Review Board (IRB) of Ewha Womans University, as the study did not involve the participation of human or animal subjects and thus posed no ethical concerns typically associated with clinical research involving living subjects (IRB No. 2024-06-051). This study was exempted from IRB, allowing the study to be conducted without additional ethical review protocols.

## 3. Results

ChatGPT provided answers to questions about frozen shoulder as follows. Question 1 was about what frozen shoulder is, and ChatGPT concisely summarized the stages of progression, causes, symptoms, diagnosis, and treatment of the condition. Question 2 was about the causes of frozen shoulder, and ChatGPT explained common causes such as age, gender, immobilization, diabetes, thyroid disorders, and inflammatory diseases. It provided relatively broad information about the factors that can lead to frozen shoulder, along with an overview of its pathophysiology. Question 3 was about the risk factors for frozen shoulder, and ChatGPT presented a broad and diverse range of risk factors, including age, gender, chronic diseases, cardiovascular disease, neurological conditions, autoimmune diseases, genetic factors, and lifestyle factors. Regarding the symptoms of frozen shoulder (Question 4), ChatGPT provided detailed descriptions of symptoms in each stage—freezing, frozen, and thawing. It described symptoms thoroughly, including less common ones like muscle weakness and sleep disturbances. Question 5 was related to the diagnosis of frozen shoulder and the answer included the information about medical history, physical examination, imaging tests, diagnostic criteria, and differential diagnosis.

ChatGPT also provided answers for the prognosis and various treatment methods for adhesive capsulitis. Question 6 was about treatment options, and it described both non-surgical treatments, such as physical therapy and medication, as well as surgical options. It also briefly described lifestyle remedies, adjunctive treatment methods, and prognosis. Question 7 was about recovery from frozen shoulder, and the response provided detailed duration estimates for each stage. It also discussed the overall recovery time and factors that may influence recovery. Question 8 was about various exercise methods for treating frozen shoulder, and the response included commonly recommended exercises for range of motion, such as the pendulum stretch, towel stretch, and finger walk. It also suggested a variety of stretching and strengthening exercises. These exercises were described clearly and concisely, making it easy for people to learn about them.

Furthermore, Question 9 was about which injection therapies can be applied for frozen shoulder, and the response included various injection methods such as steroid injections, hydrodilatation, PRP injections, and local anesthetic injections. Regarding the number of injections (Question 10), ChatGPT recommended that injection therapies be limited to 2–3 times within a 6-month period. It also included factors that may influence the number of injections. Question 11 was about the impact of frozen shoulder on daily life, and ChatGPT’s answer described in detail how it can affect activities of daily living, such as dressing, grooming, and bathing, as well as household tasks, recreational activities, sleep, and social interactions. To prevent frozen shoulder (Question 12), ChatGPT suggested regular shoulder exercises, early mobilization after surgery, managing systemic diseases, and lifestyle modifications. Question 13 was about the recurrence of frozen shoulder, and ChatGPT provided specific figures, such as a recurrence rate of 5–10%. It also detailed the risk factors associated with recurrence and methods for prevention. Lastly, Question 14 addressed when surgery is considered for frozen shoulder. It explained that surgery is typically considered in cases of prolonged symptoms, severe pain or stiffness, or the presence of other conditions. ChatGPT also provided relatively specific details on the types of surgeries performed, such as manipulation under anesthesia and arthroscopic capsular release.

After a thorough review, the authors evaluated the ChatGPT answers. Table 1 summarizes the scores of the 14 responses generated by ChatGPT, which were evaluated by rehabilitation medicine physicians using a 5-point Likert scale. The responses from ChatGPT can be accessed at https://chatgpt.com/share/ed83219f-18c4-4674-9f60-14bf704c5432 (accessed on 7 February 2025) and are included as Supplementary Data S1. In terms of validity, 12 out of 14 questions (85.7%) were rated 5 points, with questions 5 and 6 scoring 4 points. For safety, all questions except one (question 10) received a score of 5, resulting in 92.9% of questions being rated 5 points. Regarding utility, 12 out of 14 questions (85.7%) were rated 5 points, with questions 5 and 6 scoring 4 points. Considering the validity, safety, and utility of the responses, the answers provided by ChatGPT were generally rated highly.

Table 2 presents a detailed summary of the scores given to 14 ChatGPT responses, evaluated by orthopedic surgeons using a 5-point Likert scale. The evaluation of ChatGPT’s responses showed results consistent with those assessed by rehabilitation medicine physicians. In terms of validity, 12 out of 14 questions (85.7%) received the highest score of 5 points, with questions 5 and 6 scoring 4 points. For safety, 13 out of 14 questions (92.9%) were rated 5 points, with question 10 receiving a slightly lower score of 4 points. Regarding utility, 13 out of 14 questions (92.9%) were also rated 5 points, with question 6 scoring 4 points. ChatGPT’s responses were generally rated highly, demonstrating strong validity, safety, and utility, and aligning closely with the findings of the rehabilitation medicine physicians.

In question 5 (How is frozen shoulder diagnosed?), the validity and utility scores were both rated as 4 by rehabilitation medicine physicians. Orthopedic surgeons evaluated the responses generated by ChatGPT, assigning a score of 4 for validity, while rating both utility and safety with a score of 5 (Table 2). The answer lacked a detailed description of the extent of range of motion limitation that could suspect frozen shoulder, nor did it detail the specific tests that could assist in the diagnosis of frozen shoulder. In addition, the answer did not describe the typical findings of ultrasound or MRI that are commonly observed in patients with frozen shoulder.

The validity and utility scores on question 6 (What treatment options are available for frozen shoulder?) were rated 4 points because the response failed to mention the various treatment options available for treating frozen shoulder, such as hot packs, infrared radiation, shockwave diathermy, ultrasound therapy, laser therapy, extracorporeal shockwave therapy (ESWT), and manual therapy, which have been reported to be effective in treating frozen shoulder.

For question 10 (How many steroid injections are recommended for patients with frozen shoulder?), the safety score was rated 4 points. The reason for the deduction in the safety category is that it did not mention various side effects of steroid injections. In addition, although it is mentioned that caution is needed when administering steroid injections to diabetic patients (patients with conditions like diabetes may need careful monitoring due to potential effects on blood sugar levels), it does not explain why monitoring is needed and what to do to prevent detrimental effects.

## 4. Discussion

In this study, we evaluated the validity, safety, and utility of ChatGPT for providing information to patients with frozen shoulder. Among the 14 most frequently asked questions by these patients, ChatGPT provided completely reliable and comprehensive information for 85.7% of the questions. Additionally, 92.9% of the answers were deemed to pose no danger, and 85.7% were considered entirely useful. While not all answers received a perfect score, they were all rated as having relatively satisfactory validity, safety, and utility, each scoring a 4 or more. Therefore, we conclude that ChatGPT’s responses to questions about frozen shoulder are generally valid, safe, and useful.

Some questions (question 5, 6, and 10) received relatively lower scores than other questions due to the following reasons. There was a lack of information about how frozen shoulder was diagnosed in clinical practice (question 5). On physical examination, frozen shoulder is characterized by restricted active and passive ROM in at least two planes. While there is no definite consensus on the specific limitations for frozen shoulder, it may be suspected when forward flexion is less than 100°, external rotation is less than 10°, and internal rotation is limited below the L5 vertebral level [26,27]. Frozen shoulder may be diagnosed if passive external rotation is restricted to less than 50% of the opposite shoulder. Providing a simple description of ROM restrictions can help patients understand if they might have a frozen shoulder [28]. Additionally, there was an insufficient description of physical examination tests, such as the coracoid pain test [4,7,29] and Apley scratch test [30], that would help in determining the presence of frozen shoulder. The description of MRI and ultrasound findings also lacked detailed characteristics. Ultrasound (US) imaging has recently emerged as a diagnostic tool, commonly identifying the thickening of the coracohumeral ligament, axillary pouch, and rotator interval joint capsule, in addition to the obliteration of the subcoracoid fat triangle and capsular thickening [2,4,7]. Findings of MRI features, such as rotator interval and axillary joint capsule enhancement and the intensity of the inferior glenohumeral ligament, were also reported to show high sensitivities and specificities for diagnosing frozen shoulder [31].

ChatGPT did not provide sufficient information about therapeutic options for frozen shoulder and failed to provide detailed information about effective treatment methods (question 6). Previous studies have strongly recommended ESWT and laser therapy for pain relief and increasing the range of motion [32,33,34]. The application of ESWT is effective in treating patients with frozen shoulder, as it stimulates the inflammatory-mediated healing process, enhances local blood circulation, increases the extensibility of collagen fibers, and improves the range of motion [32]. High-intensity laser therapy has been reported to improve pain and quality of life in patients with frozen shoulder. It can penetrate deep into the joints and muscles, inducing analgesic, anti-inflammatory, and anti-edematous effects by reducing inflammatory mediators and enhancing circulation [33,34]. The application of various manual therapy techniques, including Kaltenborn mobilization, scapular mobilization, and manual muscle release, has also been reported to be effective in pain relief and improvement in range of motion [8,9]. ChatGPT’s omission of certain therapies, such as extracorporeal shockwave therapy (ESWT) and manual therapy, highlights its current limitations in providing comprehensive treatment guidance. While these therapies are evidence-based and have demonstrated efficacy in specific patient populations, they are generally considered complementary to foundational treatments like physical therapy, NSAIDs, and corticosteroid injections.

Regarding the safety of steroid injections (question 10), it has been reported that intra-articular steroid injections can cause various side effects such as pain, headache, rash, flushing, menstrual irregularity, atrophy of the subcutaneous fat tissue, tendon rupture, increased infection rate, weight gain, and avascular necrosis of the bone [35]. A high dose of steroid injection can increase the likelihood of these side effects, and it has been reported that patients who received a high-dose steroid injection showed significantly higher mean blood sugar levels at 6 weeks compared to those with a low dose [36]. The response lacked sufficient warnings and detailed explanations regarding this issue, as steroid injections can significantly impede blood sugar control in these individuals. Patients with diabetes may not anticipate that steroid injections for frozen shoulder can impact their glucose levels, so providing warnings is necessary.

The differentiation between scores of 4 and 5, particularly for validity and utility, reflects the level of completeness in ChatGPT’s responses. For example, in Question 5, while the response was accurate, it lacked details about diagnostic imaging findings and specific physical tests, resulting in a validity score of 4. In Question 6, the omission of adjunctive therapies like ESWT limited the utility of the response, leading to a score of 4. This scoring framework ensured consistent evaluation and highlighted areas where ChatGPT provided partially complete information. Future studies could refine this framework by adopting more granular scoring rubrics or incorporating inter-rater reliability assessments to further validate scoring consistency.

A recent meta-analysis reported that there were no clinically important differences between low-dose and high-dose steroid injections (triamcinolone acetonide 20 mg vs. 40 mg) and recommended a low-dose steroid injection considering these side effects [36]. For diabetic patients, lower doses of corticosteroids should be considered, as high doses can elevate short-term glucose levels. It would be helpful to provide patients with detailed information about the importance of the steroid dosage and make them aware of the various side effects of steroid injections. For high-risk patients, such as those with diabetes or immunosuppressive conditions, steroid injection recommendations must be paired with clear warnings emphasizing the need for consultation with a healthcare professional.

The findings of the present study align with the existing literature emphasizing the potential of AI tools like ChatGPT to promote user engagement and understanding in both healthcare and educational settings. Shahsavar and Choudhury [37] demonstrated that even though ChatGPT is not specifically designed for healthcare management, people who are familiar with ChatGPT tend to ask questions related to health conditions. Similarly, the study by Davy Tsz Kit Ng et al. [38] highlights the effectiveness of generative AI technologies in promoting self-regulated learning through personalized feedback and adaptable responses. Applying these insights to healthcare, ChatGPT could be further refined to support patient self-education by encouraging proactive engagement with health information, empowering improved health literacy and decision-making. By integrating self-regulated learning principles and tailoring responses to individual contexts, ChatGPT appears to provide reliable and verified answers to patients with frozen shoulder, which can help increase their awareness of the disease. Also, ChatGPT has the potential to serve as a more effective tool for patient education and engagement. However, ensuring its safe and effective use requires collaboration among healthcare providers, AI developers, and policy-makers to regularly update its knowledge base, validate its outputs, and enhance transparency about its limitations.

Overall, ChatGPT provided useful information about what frozen shoulder is, as well as how it is diagnosed and treated. Some questions did not receive a full score in validity, safety, and utility. However, in clinical practice, it is challenging for physicians to provide all information in response to patients’ questions. In addition, there is still no clear consensus on a single effective treatment method to fully cure frozen shoulder. The information provided by ChatGPT was overall very useful and seems to greatly help patients understand this condition.

The limitations of this study are as follows. First, ChatGPT did not seem to include up-to-date information on the diagnosis and treatment of frozen shoulder. Second, the data that ChatGPT was trained on may have been biased or outdated. Patients should be aware that the accuracy of ChatGPT’s answers may decrease when there is only limited published information. Additionally, the answers provided by ChatGPT may vary at different times. It is also uncertain whether people without a medical background would understand the answers given by ChatGPT. For the safety-related question, our primary focus was on evaluating whether the provided information could pose any harm to patients. However, a thorough safety assessment should also encompass the broader clinical context, including patient comorbidities and concurrent treatments. Also, the use of a 5-point Likert scale by evaluators introduces some degree of subjectivity. Although the evaluators in this study are experienced clinicians, the results could vary if assessed by different individuals. Future research should include inter-rater reliability analysis or expand the number of evaluators to enhance consistency and reduce bias. It is also important to note that the concept of recent or outdated data does not inherently apply to ChatGPT’s training dataset, as it consists of a diverse, static collection of information up to a specific cutoff date. As such, users should critically evaluate all outputs, avoiding uncritical reliance on the information provided. By recognizing these limitations, researchers and practitioners can better contextualize AI-generated outputs and mitigate potential risks associated with outdated or incomplete data. Additionally, this study used ChatGPT 4.0 at a specific time point, and we did not track longitudinal changes. Further studies that include monitoring AI updates and assessing consistency over time are needed in the future. A notable limitation of this study is that responses generated by ChatGPT were collected at a single time point, and no repeated attempts were made to evaluate consistency across multiple trials. This decision was guided by the exploratory nature of the study, which aimed to assess the feasibility and initial validity of utilizing ChatGPT in the given context. Future studies should address this limitation by incorporating repeated trials across different time points and conditions. Another limitation of ChatGPT is its inability to provide references or source citations for its recommendations. This limits the ability of users to directly verify the alignment of its responses with current clinical guidelines or evidence-based practices. In this study, we mitigated this limitation by relying on expert clinicians to evaluate the content; however, this approach is inherently subjective. Future versions of ChatGPT and similar AI tools would benefit greatly from integrating explicit references into their responses. This would enhance the model’s transparency and allow users to trace the provided information back to its source, ensuring better alignment with clinical standards and evidence-based medicine. Finally, ChatGPT’s responses are fundamentally general, as the model does not consider individual patient circumstances, preferences, or cultural sensitivities. This limitation may result in less relevant or non-specific advice for certain users. Future studies could explore methods to incorporate patient-specific variables to enhance the relevance and applicability of AI-generated information. Further appropriate strategies to minimize potential risks and negative outcomes are needed in the future. Another notable limitation of ChatGPT is the variability of its responses, which can confuse users without a medical background. Usage guidelines encourage users to frame clear, specific queries and cross-check information with reliable sources.

Responses to questions such as “How is frozen shoulder diagnosed?” can be improved by incorporating standardized diagnostic criteria into the model’s training data. For example, including specific details about range of motion (ROM) limitations, validated clinical tests (e.g., coracoid pain test, Apley scratch test), and imaging findings (e.g., thickening of the coracohumeral ligament on ultrasound or enhancement of the rotator interval on MRI) could provide users with more robust and practical guidance. Integrating structured medical databases, such as PubMed, alongside up-to-date diagnostic protocols would be an effective approach to achieving this improvement. Additionally, enabling ChatGPT to provide patient-specific recommendations requires training the model on diverse clinical case scenarios. For instance, answering questions like “How many steroid injections are recommended?” with tailored warnings for specific patient groups, such as those with diabetes, would necessitate the AI recognizing risk factors and adjusting its guidance accordingly. This could be achieved by developing algorithms that dynamically modify responses based on patient profiles or by incorporating datasets emphasizing condition-specific risks and management strategies. Currently, ChatGPT’s responses lack information on evidence-based adjunctive therapies, such as extracorporeal shockwave therapy (ESWT) or high-intensity laser therapy, which have demonstrated efficacy in clinical trials. Addressing this gap would require routine updates to the model’s training data with curated, peer-reviewed literature, clinical practice guidelines, and meta-analyses. Furthermore, establishing partnerships with medical organizations to provide continuous input on advancements in treatment modalities would further enhance the model’s scope and accuracy. By incorporating these features, AI models like ChatGPT could provide more comprehensive, patient-specific, and up-to-date guidance, thereby increasing their utility in both patient education and clinical practice. Such improvements would require ongoing collaboration among AI developers, clinicians, and medical organizations to ensure alignment with the latest advancements in healthcare.

ChatGPT’s responses are generated based on training data and may not reflect the most up-to-date medical guidelines or research. Therefore, users should consult a qualified healthcare provider for any questions or concerns regarding their medical condition or treatment options. Patients may use ChatGPT as a supplementary tool rather than the sole source of medical information.

## 5. Conclusions

In conclusion, ChatGPT demonstrates moderate validity, safety, and usefulness in providing information for patients with frozen shoulder. Its advantage lies in its ability to instantly answer questions related to medical conditions, and any person without a medical background can use ChatGPT anytime, anywhere. ChatGPT’s responses should be used as a supplementary tool for patient education and not as a replacement for professional medical advice, diagnosis, or treatment. Users must be aware of its limitations, including the potential for incomplete or erroneous responses, outdated information, and the absence of individualized guidance. If unaddressed, these limitations pose potential risks to patient health, particularly when AI-generated information is used without expert validation. To safely integrate ChatGPT into patient care, healthcare providers and users should cross-check its recommendations with up-to-date clinical guidelines. When used responsibly, ChatGPT can serve as a valuable resource to enhance patient education and engagement, but it should always be complemented by professional medical expertise.

## Figures and Tables

**Table 1:** Evaluation of ChatGPT responses using a 5-point Likert scale by rehabilitation medicine physicians.

Questions	Validity	Safety	Utility	Total
What is frozen shoulder (adhesive capsulitis)?	5	5	5	15
2. What causes frozen shoulder?	5	5	5	15
3. What risk factors or conditions increase the likelihood of developing frozen shoulder?	5	5	5	15
4. What are the common symptoms of frozen shoulder?	5	5	5	15
5. How is frozen shoulder diagnosed?	4	5	4	13
6. What treatment options are available for frozen shoulder?	4	5	4	13
7. How long does recovery from frozen shoulder typically take?	5	5	5	15
8. What exercises are recommended for managing frozen shoulder?	5	5	5	15
9. What types of injections are available for patients with frozen shoulder?	5	5	5	15
10. How many steroid injections are recommended for patients with frozen shoulder?	5	4	5	14
11. What effect does frozen shoulder have on everyday activities?	5	5	5	15
12. What measures can be taken to prevent adhesive capsulitis?	5	5	5	15
13. How often does frozen recur?	5	5	5	15
14. Under what circumstances should surgery be considered for treating frozen shoulder?	5	5	5	15

**Table 2:** Evaluation of ChatGPT responses using a 5-point Likert scale by orthopedic surgeons.

Questions	Validity	Safety	Utility	Total
What is frozen shoulder (adhesive capsulitis)?	5	5	5	15
2. What causes frozen shoulder?	5	5	5	15
3. What risk factors or conditions increase the likelihood of developing frozen shoulder?	5	5	5	15
4. What are the common symptoms of frozen shoulder?	5	5	5	15
5. How is frozen shoulder diagnosed?	4	5	5	13
6. What treatment options are available for frozen shoulder?	4	5	4	13
7. How long does recovery from frozen shoulder typically take?	5	5	5	15
8. What exercises are recommended for managing frozen shoulder?	5	5	5	15
9. What types of injections are available for patients with frozen shoulder?	5	5	5	15
10. How many steroid injections are recommended for patients with frozen shoulder?	5	4	5	14
11. What effect does frozen shoulder have on everyday activities?	5	5	5	15
12. What measures can be taken to prevent adhesive capsulitis?	5	5	5	15
13. How often does frozen recur?	5	5	5	15
14. Under what circumstances should surgery be considered for treating frozen shoulder?	5	5	5	15

## Data Availability

Data are available in the Supplementary Materials.

## Supplementary Materials

The following supporting information can be downloaded at: https://www.mdpi.com/article/10.3390/life15020262/s1, Data S1: The detailed answers of ChatGPT for frozen shoulder.
