# Supplementary material for: Assessing the Validity, Safety, and Utility of ChatGPT’s Responses for Patients with Frozen Shoulder
Source: Life (Basel). 2025 Feb 8;15(2):262. doi: 10.3390/life15020262 (PMC11857066; doi:10.3390/life15020262)
Supplement: Supplementary file 1 [file life-15-00262-s001.zip › life-3406289-supplementary.pdf]

Supplementary Data S1. The detailed answers of ChatGPT for frozen shoulder

1. What is frozen shoulder (adhesive capsulitis)?

Frozen shoulder, also known as adhesive capsulitis, is a condition characterized by stiffness and pain in the shoulder joint. It occurs when the connective tissue surrounding the shoulder joint, known as the shoulder capsule, thickens and tightens around the joint, restricting its movement. This condition typically develops gradually and can be classified into three stages:

1. **Freezing Stage**: During this initial stage, any movement of the shoulder causes pain, and the shoulder's range of motion starts to become limited. This stage can last from six weeks to nine months.
2. **Frozen Stage**: Pain may begin to diminish during this stage, but the shoulder becomes stiffer, and using it becomes more difficult. This stage can last from four to twelve months.
3. **Thawing Stage**: The range of motion in the shoulder begins to improve during this stage. Complete return to normal or close-to-normal strength and movement typically occurs. This stage can last from six months to two years.

Causes

The exact cause of frozen shoulder is not well understood, but several factors may increase the risk of developing it:

- **Age and Gender**: It is more common in people between the ages of 40 and 60, and it affects women more often than men.
- **Prolonged Immobilization**: A period of immobility due to surgery, injury, or another shoulder problem can lead to the development of frozen shoulder.
- **Systemic Diseases**: Certain systemic conditions, such as diabetes, thyroid disorders, cardiovascular disease, and Parkinson's disease, are associated with an increased risk of developing frozen shoulder.

Symptoms

- Pain and stiffness in the shoulder
- Limited range of motion
- Difficulty performing daily activities that involve shoulder movement

Diagnosis

Diagnosis is usually made based on medical history, a physical examination, and imaging tests such as X-rays or MRI to rule out other shoulder conditions.

Treatment

Treatment for frozen shoulder focuses on relieving pain and restoring the shoulder's range of motion. Options include:

- **Physical Therapy**: Exercises and stretches to improve flexibility and range of motion.
- **Medications**: Pain relievers and anti-inflammatory drugs.
- **Steroid Injections**: Corticosteroid injections can help reduce pain and inflammation.
- **Shoulder Manipulation**: In severe cases, under anesthesia, the shoulder is moved to help loosen the tightened tissue.
- **Surgery**: Rarely, arthroscopic surgery is performed to remove scar tissue and adhesions.

Most people with frozen shoulder regain almost all their shoulder motion and function with time and appropriate treatment.

2. What causes frozen shoulder?

The exact cause of frozen shoulder (adhesive capsulitis) is not fully understood, but several factors and conditions are known to increase the risk of developing it. These include:

1. **Immobilization**: Prolonged immobility of the shoulder due to surgery, injury, or another shoulder condition can lead to frozen shoulder. For example, people who have had a stroke, broken arm, or surgery are more likely to develop it.
2. **Systemic Diseases**:
   - **Diabetes**: People with diabetes are at a higher risk. The reasons for this association are unclear, but it is estimated that about 10-20% of people with diabetes will develop frozen shoulder.
   - **Thyroid Disorders**: Conditions like hypothyroidism and hyperthyroidism are linked to an increased risk.
   - **Cardiovascular Disease**: Heart disease and other cardiovascular conditions may contribute to the development of frozen shoulder.
   - **Parkinson’s Disease**: Neurological conditions like Parkinson’s disease are also associated with an increased risk.
3. **Age and Gender**: It is more common in people between the ages of 40 and 60 and affects women more often than men.
4. **Other Shoulder Conditions**: Conditions like rotator cuff injuries, tendinitis, and bursitis that limit shoulder movement can also contribute to the development of frozen shoulder.
5. **Inflammation and Autoimmune Response**: Chronic inflammation in the shoulder joint and an abnormal autoimmune response may play a role in the development of adhesive capsulitis, although this is not fully understood.
6. **Genetics**: There may be a genetic predisposition, as it sometimes runs in families.

Pathophysiology

The underlying process involves the thickening and contraction of the shoulder joint capsule and the formation of adhesions. The capsule, which is a thick layer of connective tissue that surrounds the shoulder joint, becomes inflamed and scarred, leading to stiffness and pain.

Prevention

While it's not always possible to prevent frozen shoulder, certain measures can reduce the risk:

- Keeping the shoulder joint active and avoiding prolonged immobilization.
- Performing gentle range-of-motion exercises after surgery or an injury.
- Managing underlying health conditions such as diabetes and thyroid disorders effectively.

Understanding these risk factors and early intervention can help in managing and potentially preventing the development of frozen shoulder.

3. What risk factors or conditions increase the likelihood of developing frozen shoulder?

Several risk factors and conditions increase the likelihood of developing frozen shoulder (adhesive capsulitis). These include:

1. **Age and Gender**:
    - People aged 40 to 60 are more likely to develop frozen shoulder.
    - Women are more commonly affected than men.
2. **Diabetes**:
    - Individuals with diabetes have a significantly higher risk. It's estimated that about 10-20% of people with diabetes will develop frozen shoulder.
    - The exact reasons for this association are unclear, but it may be related to changes in collagen or glycosylation processes.
3. **Thyroid Disorders**:
    - Conditions such as hypothyroidism and hyperthyroidism are linked to an increased risk of frozen shoulder.
4. **Cardiovascular Disease**:
    - Heart disease and other cardiovascular conditions have been associated with an increased risk of developing frozen shoulder.
5. **Neurological Conditions**:
    - Diseases like Parkinson’s disease increase the likelihood of frozen shoulder due to potential changes in muscle tone and movement.
6. **Prolonged Immobilization**:
    - Immobilization of the shoulder due to surgery, injury (such as a broken arm), or another shoulder problem can lead to frozen shoulder. This immobilization may result from a fracture, stroke, or post-surgical recovery.

7. **Other Shoulder Conditions**:
    - Conditions such as rotator cuff injuries, tendinitis, and bursitis that limit shoulder movement can contribute to the development of frozen shoulder.
8. **Autoimmune and Inflammatory Diseases**:
    - Chronic inflammation in the shoulder joint and autoimmune responses may play a role in the development of adhesive capsulitis.
9. **Genetic Factors**:
    - There may be a genetic predisposition, as frozen shoulder sometimes runs in families.
10. **Lifestyle Factors**:

- Sedentary lifestyle or lack of regular shoulder movement may contribute to the development of this condition.

Understanding these risk factors can help in identifying individuals at higher risk and implementing preventive measures, such as maintaining shoulder mobility through regular exercise and effectively managing underlying health conditions.

4. What are the common symptoms of frozen shoulder?

The common symptoms of frozen shoulder (adhesive capsulitis) typically develop gradually and can be categorized into three stages: the freezing stage, the frozen stage, and the thawing stage. Here are the detailed symptoms associated with each stage:

Freezing Stage

- **Pain**: Persistent pain in the shoulder, which can be severe and worsen at night.
- **Stiffness**: Gradual onset of stiffness in the shoulder, making it difficult to move the joint.
- **Limited Range of Motion**: Progressive loss of shoulder movement, especially with activities that involve lifting the arm or reaching behind the back.
- **Duration**: This stage can last from six weeks to nine months.

Frozen Stage

- **Reduced Pain**: Pain may start to decrease during this stage, particularly during rest.
- **Increased Stiffness**: The shoulder becomes increasingly stiff, leading to a significant reduction in the range of motion.
- **Difficulty with Daily Activities**: Activities that require shoulder movement become challenging, such as dressing, reaching for objects, or performing overhead tasks.
- **Duration**: This stage can last from four to twelve months.

Thawing Stage

- **Improvement in Range of Motion**: Gradual improvement in the shoulder's range of motion and a decrease in stiffness.
- **Minimal Pain**: Pain continues to diminish and may be completely absent by the end of this stage.

- **Return of Function**: Slow but steady return of shoulder function, allowing for more normal activities.
- **Duration**: This stage can last from six months to two years.

General Symptoms

- **Pain and Discomfort**: Generalized pain in the shoulder, which may radiate down the arm.
- **Muscle Weakness**: Due to reduced use of the shoulder, the muscles around the joint may weaken.
- **Sleep Disturbance**: Nighttime pain can interfere with sleep.

Frozen shoulder typically resolves over time, but the duration and severity of symptoms can vary. Early diagnosis and treatment, including physical therapy and appropriate pain management, can help reduce symptoms and improve the shoulder's range of motion.

5. How is frozen shoulder diagnosed?

Diagnosing frozen shoulder (adhesive capsulitis) involves a combination of medical history, physical examination, and imaging tests. Here are the detailed steps typically taken to diagnose this condition:

Medical History

The doctor will start by asking about the patient's medical history, including:

- The onset and duration of symptoms.
- The nature and severity of pain.
- Any history of shoulder injuries or surgeries.
- The presence of underlying conditions such as diabetes, thyroid disorders, or cardiovascular diseases.
- Any recent periods of shoulder immobilization or decreased use.

Physical Examination

During the physical examination, the doctor will:

- **Assess Range of Motion**: The doctor will evaluate both active (patient moves the shoulder) and passive (doctor moves the shoulder) range of motion. In frozen shoulder, both active and passive ranges of motion are typically reduced.
- **Check for Pain and Tenderness**: The doctor will palpate the shoulder and ask the patient to move it in different directions to identify areas of pain and tenderness.
- **Compare Shoulders**: The doctor may compare the affected shoulder with the unaffected one to assess the extent of stiffness and restriction.

Imaging Tests

To rule out other shoulder conditions, such as arthritis or rotator cuff injuries, the doctor may order imaging tests:

- **X-rays**: While X-rays cannot show frozen shoulder directly, they can help rule out other causes of shoulder pain and stiffness, such as arthritis or bone abnormalities.
- **MRI (Magnetic Resonance Imaging)** or **Ultrasound**: These imaging techniques can provide detailed images of the soft tissues around the shoulder joint. They help identify issues like rotator cuff tears or inflammation that may mimic the symptoms of frozen shoulder.

Diagnosis Criteria

The diagnosis of frozen shoulder is generally based on the following criteria:

- **History of gradual onset shoulder pain and stiffness**.
- **Significant reduction in both active and passive range of motion**.
- **Normal or near-normal X-rays** (to rule out other conditions).

Differential Diagnosis

The doctor will also consider other conditions that can cause similar symptoms, such as:

- Rotator cuff tears
- Shoulder arthritis
- Tendinitis
- Bursitis

Additional Tests

In some cases, additional tests might be conducted to rule out systemic diseases associated with frozen shoulder, such as blood tests for diabetes or thyroid function.

Early and accurate diagnosis is essential for managing frozen shoulder effectively, as it allows for timely intervention and appropriate treatment to reduce symptoms and improve shoulder function.

6. What treatment options are available for frozen shoulder?

Treatment for frozen shoulder (adhesive capsulitis) aims to relieve pain and restore the shoulder's range of motion. The treatment options can be categorized into non-surgical and surgical approaches, depending on the severity and duration of the condition.

Non-Surgical Treatments

1. **Physical Therapy**:
   - **Stretching Exercises**: Specific exercises to improve flexibility and range of motion.
   - **Strengthening Exercises**: Once pain allows, exercises to strengthen the shoulder muscles.
   - **Manual Therapy**: Techniques used by a physical therapist to mobilize the shoulder joint.
2. **Medications**:

   - **Pain Relievers**: Over-the-counter medications like acetaminophen or nonsteroidal anti-inflammatory drugs (NSAIDs) such as ibuprofen or naproxen to reduce pain and inflammation.
   - **Corticosteroids**: Oral or injectable corticosteroids to reduce inflammation and pain. These can be particularly helpful in the early stages.
3. **Hot and Cold Therapy**:
   - Applying heat or ice packs to the shoulder to manage pain and inflammation.
4. **Hydrodilatation (Distension Arthrography)**:
   - This procedure involves injecting a large volume of sterile fluid into the shoulder joint to stretch the capsule and improve range of motion.

Surgical Treatments

1. **Manipulation Under Anesthesia (MUA)**:
   - While the patient is under general anesthesia, the doctor forcibly moves the shoulder to break up the adhesions and scar tissue. This procedure is generally reserved for severe cases where non-surgical treatments have failed.
2. **Arthroscopic Surgery**:
   - A minimally invasive procedure where a small camera (arthroscope) and surgical instruments are inserted through tiny incisions to cut through the thickened, tight parts of the joint capsule. This procedure is considered when other treatments have not provided sufficient relief.

Adjunctive Treatments

1. **Steroid Injections**:
   - Injections of corticosteroids directly into the shoulder joint to reduce inflammation and pain.
2. **Transcutaneous Electrical Nerve Stimulation (TENS)**:
   - A device that uses low-voltage electrical currents to relieve pain.

Lifestyle and Home Remedies

1. **Activity Modification**:
   - Avoid activities that exacerbate the pain, but keep the shoulder as mobile as possible with gentle movements and exercises.
2. **Home Exercises**:
   - Performing prescribed stretching and range-of-motion exercises at home to maintain and improve flexibility.

Preventive Measures

1. **Early Mobilization**:
   - After any shoulder injury or surgery, it's important to start gentle movements and physical therapy as soon as recommended to prevent the development of frozen shoulder.
2. **Managing Underlying Conditions**:
   - Keeping chronic conditions like diabetes and thyroid disorders well-controlled can reduce the risk of developing frozen shoulder.

Prognosis

Most people with frozen shoulder recover fully with appropriate treatment, although it may take several months to years. Early diagnosis and intervention can significantly improve outcomes and reduce the duration of symptoms.

7. How long does recovery from frozen shoulder typically take?

Recovery from frozen shoulder (adhesive capsulitis) typically occurs over a prolonged period and can be divided into three stages: the freezing stage, the frozen stage, and the thawing stage. The total duration of recovery can vary significantly among individuals but generally falls within the following range:

Stages of Recovery

1. **Freezing Stage**:
   - **Duration**: This initial stage can last from six weeks to nine months.
   - **Symptoms**: Gradual onset of pain and stiffness. The range of motion becomes increasingly limited.
2. **Frozen Stage**:
   - **Duration**: This stage can last from four to twelve months.
   - **Symptoms**: Pain may decrease, but the shoulder remains very stiff. Daily activities involving shoulder movement become more difficult.
3. **Thawing Stage**:
   - **Duration**: This stage can last from six months to two years.
   - **Symptoms**: Gradual improvement in the range of motion. Pain continues to diminish, and shoulder function slowly returns to normal or near-normal levels.

Overall Recovery Time

The entire process from the onset of symptoms to complete recovery typically takes between one to three years. However, the exact duration can vary based on several factors, including the severity of the condition, the effectiveness of the treatment, and the individual's overall health and compliance with physical therapy.

Factors Influencing Recovery Time

- **Severity of the Condition**: More severe cases with significant stiffness and pain may take longer to recover.
- **Early Diagnosis and Treatment**: Early intervention with physical therapy and other treatments can help shorten the duration of symptoms.
- **Underlying Health Conditions**: Conditions like diabetes or thyroid disorders can prolong recovery.
- **Adherence to Treatment**: Consistent participation in physical therapy and home exercises can significantly impact recovery time.
- **Individual Variability**: Each person's response to treatment and recovery process can differ.

Summary

While frozen shoulder can be a lengthy condition to recover from, most people eventually regain almost all their shoulder function. It's important to follow the prescribed treatment plan, stay active within pain limits, and have regular follow-ups with a healthcare provider to monitor progress and adjust treatments as necessary.

8. What exercises are recommended for managing frozen shoulder?

Managing frozen shoulder (adhesive capsulitis) involves exercises that help improve flexibility, restore range of motion, and strengthen the shoulder muscles. These exercises should be performed gently and consistently, usually under the guidance of a physical therapist. Here are some commonly recommended exercises for frozen shoulder:

Range of Motion Exercises

1. **Pendulum Stretch**:
    - Lean forward slightly, allowing the affected arm to hang down.
    - Swing the arm in small circles, clockwise and counterclockwise, for about 10 times in each direction.
    - Gradually increase the diameter of the circles as the pain allows.
2. **Towel Stretch**:
    - Hold one end of a towel behind your back and grab the opposite end with the other hand.
    - Use the good arm to pull the affected arm upward to stretch it.
    - Hold for 10-20 seconds and repeat 10-20 times a day.
3. **Finger Walk**:
    - Stand facing a wall at arm's length.
    - With the affected arm, use your fingers to "walk" up the wall, spider-like, as far as you comfortably can.
    - Keep your elbow slightly bent and hold each position for a few seconds before walking your fingers back down.
    - Repeat 10-20 times a day.
4. **Cross-Body Reach**:
    - Use your good arm to lift the affected arm at the elbow and bring it up and across your body, exerting gentle pressure to stretch the shoulder.
    - Hold for 15-20 seconds.
    - Repeat 10-20 times a day.

Stretching Exercises

1. **Armpit Stretch**:
    - Using your good arm, lift the affected arm onto a shelf about breast-high.
    - Gently bend your knees, opening up the armpit.
    - Deepen the bend in your knees slightly, stretching the armpit, and then straighten.
    - With each knee bend, stretch a little further, but be gentle.
    - Repeat 10-20 times a day.
2. **External Rotation**:
    - Hold a resistance band between your hands with your elbows at a 90-degree angle close to your sides.
    - Rotate the affected arm outward two to three inches and hold for five seconds.

    - Repeat 10-15 times a day.

3. **Internal Rotation**:
    - Stand next to a closed door, and hook one end of a resistance band around the doorknob.
    - Hold the other end with your affected arm, keeping your elbow at a 90-degree angle.
    - Pull the band towards your body, rotating your shoulder inward.
    - Hold for a few seconds and return to the starting position.
    - Repeat 10-15 times a day.

Strengthening Exercises

1. **Isometric Shoulder Exercises**:
    - **Internal Rotation**: Press the palm of the affected arm against a wall, holding for a few seconds.
    - **External Rotation**: Push the back of the affected hand against a wall, holding for a few seconds.
    - **Flexion**: Press the front of the affected arm against a wall, holding for a few seconds.
    - **Extension**: Press the back of the affected arm against a wall, holding for a few seconds.
2. **Scapular Stabilization**:
    - Perform exercises that target the muscles around the shoulder blade, such as scapular retractions (pulling the shoulder blades together) and depressions (pulling the shoulder blades downward).

Important Tips

- **Warm-Up**: Always warm up your shoulder before starting exercises. Use a warm towel or take a warm shower to loosen up the muscles.
- **Gradual Progression**: Start with gentle exercises and gradually increase the intensity and range of motion as your pain allows.
- **Consistency**: Perform the exercises regularly, as recommended by your physical therapist.
- **Avoid Overstretching**: Do not force any movements or stretch to the point of pain. Gentle stretching is key.

These exercises can help manage frozen shoulder by improving flexibility and strength while reducing pain and stiffness. Always consult with a healthcare professional or physical therapist before starting any exercise program to ensure it is appropriate for your specific condition.

9. What types of injections are available for patients with frozen shoulder?

For patients with frozen shoulder (adhesive capsulitis), various types of injections can be used to help reduce pain and inflammation, as well as to improve range of motion. Here are the most commonly used injections:

1. Corticosteroid Injections

Description: Corticosteroids are powerful anti-inflammatory medications that can reduce pain and swelling in the shoulder joint.

Procedure: The injection is typically administered directly into the shoulder joint or the surrounding soft tissues.

Benefits:

- Quick relief from pain and inflammation.
- Can help improve range of motion, particularly in the early stages of frozen shoulder.

Drawbacks:

- Relief is usually temporary, and the effect can diminish over time.
- Potential side effects include weakening of tendons and cartilage, and repeated injections can increase the risk of infection and tissue damage.

2. Hydrodilatation (Distension Arthrography)

Description: This involves injecting a large volume of sterile saline solution, often mixed with a local anesthetic and sometimes corticosteroid, into the shoulder joint.

Procedure: The injection stretches the joint capsule, which can help break adhesions and improve range of motion.

Benefits:

- Can provide significant pain relief and improve shoulder mobility.
- Often used when other treatments have not been effective.

Drawbacks:

- There may be discomfort during the procedure.
- Possible side effects include pain, swelling, and infection.

3. Platelet-Rich Plasma (PRP) Injections

Description: PRP involves injecting a concentration of the patient's own platelets, which are believed to promote healing.

Procedure: Blood is drawn from the patient, processed to concentrate the platelets, and then injected into the affected area.

Benefits:

- May help in tissue repair and reduce inflammation.
- Uses the patient's own blood, reducing the risk of adverse reactions.

Drawbacks:

- Limited evidence on the effectiveness for frozen shoulder specifically.
- Can be expensive and not always covered by insurance.

4. Local Anesthetic Injections

Description: These injections use anesthetics like lidocaine to numb the shoulder area temporarily.

Procedure: Administered directly into the shoulder joint or surrounding tissues.

Benefits:

- Provides temporary pain relief, allowing for more effective physical therapy.
- Can help confirm the diagnosis by distinguishing between different sources of shoulder pain.

Drawbacks:

- Only provides short-term relief.
- Does not address the underlying inflammation or stiffness.

Considerations and Recommendations

- **Timing and Frequency**: Injections are often used in conjunction with physical therapy. Timing and frequency should be carefully considered to maximize benefits and minimize risks.
- **Individual Response**: Patients may respond differently to each type of injection. A healthcare provider can help determine the most appropriate type based on the individual's condition and response to previous treatments.
- **Potential Side Effects**: While injections can be effective, they also carry risks such as infection, allergic reactions, and tissue damage. These risks should be discussed with a healthcare provider.

It's essential to consult with a healthcare professional to determine the most suitable injection therapy based on the specific stage and severity of frozen shoulder, as well as the patient's overall health and treatment goals.

10. How many steroid injections are recommended for patients with frozen shoulder?

The number of steroid injections recommended for patients with frozen shoulder (adhesive capsulitis) varies based on individual patient circumstances and response to treatment. Generally, healthcare providers follow these guidelines:

Typical Recommendations:

1. **Initial Injection**: Often, a single injection is given initially to assess the patient’s response.

2. **Follow-Up Injections**: If the patient experiences significant pain relief and improvement in range of motion, but symptoms persist or recur, additional injections may be considered.

General Guidelines:

- **Number of Injections**: Typically, no more than 2 to 3 steroid injections are recommended within a six-month period. This limit helps reduce the risk of potential side effects associated with repeated steroid use, such as weakening of tendons and cartilage, increased risk of infection, and tissue damage.
- **Intervals**: Injections are usually spaced several weeks to a few months apart to allow time for the steroid to take effect and to monitor the patient's response.

Factors Influencing the Number of Injections:

1. **Severity of Symptoms**: More severe pain and stiffness may warrant multiple injections, but the overall number should still be limited.
2. **Response to Initial Injection**: If the first injection provides substantial relief, subsequent injections may be more spaced out or limited.
3. **Stage of Frozen Shoulder**: Injections might be more beneficial in the early stages (freezing stage) to reduce inflammation and pain.
4. **Underlying Health Conditions**: Patients with conditions like diabetes may need careful monitoring due to potential effects on blood sugar levels.

Alternative or Adjunct Treatments:

- **Physical Therapy**: Regular physical therapy and home exercises are crucial and often used alongside injections to maximize the improvement in range of motion.
- **Other Injections**: Hydrodilatation (distension arthrography) or platelet-rich plasma (PRP) injections might be considered if steroid injections are not effective or appropriate.
- **Medications**: Oral pain relievers and anti-inflammatory drugs can also be used to manage symptoms between injections.

Summary:

While steroid injections can be effective in managing pain and improving mobility in frozen shoulder, their use should be limited to 2 to 3 injections within six months. This approach minimizes potential side effects and ensures that other treatments, particularly physical therapy, are utilized to address the underlying stiffness and improve shoulder function. Always consult with a healthcare provider to determine the most appropriate treatment plan based on individual needs and responses.

11. What effect does frozen shoulder have on everyday activities?

Frozen shoulder (adhesive capsulitis) can significantly impact everyday activities due to pain, stiffness, and reduced range of motion in the shoulder. Here are some specific ways in which it affects daily life:

1. Personal Care

- **Dressing and Undressing**: Difficulty putting on and taking off clothes, especially garments that require arm movements above the head or behind the back.
- **Grooming**: Challenges with combing or washing hair due to limited arm movement.
- **Bathing**: Difficulty reaching parts of the body, such as the back, due to shoulder stiffness.

2. Household Activities

- **Cooking and Cleaning**: Trouble lifting pots, pans, or other kitchen items, and performing cleaning tasks that require reaching or stretching.
- **Laundry**: Difficulty reaching into washers or dryers, and hanging clothes on a line or hanger.
- **Yard Work**: Challenges with gardening, raking, or other outdoor chores that require shoulder mobility.

3. Work-Related Tasks

- **Office Work**: Difficulty typing on a computer or reaching for items on a desk or shelves.
- **Manual Labor**: Inability to perform tasks that require lifting, pushing, or pulling with the affected arm.
- **Driving**: Trouble steering, shifting gears, or reaching for controls within the vehicle.

4. Recreational Activities

- **Sports**: Difficulty participating in sports that involve shoulder movements, such as swimming, tennis, or golf.
- **Hobbies**: Challenges with hobbies that require fine motor skills and shoulder mobility, such as painting, knitting, or playing a musical instrument.

5. Sleeping

- **Comfort**: Pain and stiffness can make it difficult to find a comfortable sleeping position, leading to disrupted sleep.
- **Turning**: Difficulty turning in bed due to shoulder pain and limited range of motion.

6. Social and Emotional Impact

- **Social Activities**: Limited ability to participate in social activities that require shoulder use, potentially leading to social isolation.
- **Emotional Well-Being**: Chronic pain and disability can lead to frustration, anxiety, and depression.

Coping Strategies

- **Adapting Activities**: Finding alternative ways to perform tasks, such as using assistive devices or modifying movements.
- **Pain Management**: Using pain relief methods such as medication, hot/cold therapy, and gentle exercises to manage symptoms.
- **Professional Help**: Seeking physical therapy to improve range of motion and occupational therapy to adapt daily activities.

Summary

Frozen shoulder can significantly hinder everyday activities, affecting personal care, household tasks, work, recreational activities, sleep, and social interactions. Managing these

challenges often requires a combination of medical treatment, physical therapy, and lifestyle adaptations to maintain functionality and improve quality of life.

12. What measures can be taken to prevent adhesive capsulitis?

Preventing adhesive capsulitis (frozen shoulder) involves maintaining shoulder mobility and addressing risk factors that may contribute to its development. Here are some measures that can be taken to reduce the risk:

1. Regular Shoulder Exercises

- **Range of Motion Exercises**: Engage in regular exercises that promote shoulder flexibility and range of motion, such as arm circles, shoulder rolls, and stretching exercises.
- **Strengthening Exercises**: Incorporate exercises that strengthen the shoulder muscles, such as resistance band exercises and weightlifting, to maintain joint stability and function.

2. Early Mobilization After Injury or Surgery

- **Physical Therapy**: Start physical therapy as soon as recommended after a shoulder injury or surgery to prevent stiffness.
- **Gentle Movements**: Perform gentle shoulder movements and stretches during the recovery period to maintain mobility.

3. Managing Underlying Health Conditions

- **Diabetes Control**: Keep blood sugar levels well-managed if you have diabetes, as this condition increases the risk of frozen shoulder.
- **Thyroid Health**: Monitor and manage thyroid conditions, as hypothyroidism and hyperthyroidism are associated with an increased risk.
- **Overall Health**: Address cardiovascular and other systemic health issues that may contribute to the development of frozen shoulder.

4. Avoiding Prolonged Immobilization

- **Activity Modification**: Avoid prolonged periods of shoulder immobilization. If immobilization is necessary (e.g., due to a fracture), perform gentle, physician-approved movements as soon as possible.
- **Regular Movement**: Ensure regular movement and use of the shoulder joint in daily activities to prevent stiffness.

5. Ergonomic Adjustments

- **Workstation Setup**: Ensure that your workstation is ergonomically designed to avoid prolonged strain on the shoulder. Adjust the height of your chair, desk, and computer monitor to promote good posture.
- **Frequent Breaks**: Take regular breaks from repetitive tasks to stretch and move your shoulders.

6. Lifestyle and Home Remedies

- **Hot and Cold Therapy**: Apply heat or cold packs to the shoulder to alleviate minor aches and maintain flexibility.
- **Healthy Diet and Hydration**: Maintain a balanced diet and stay hydrated to support overall joint health.

7. Regular Check-ups

- **Medical Monitoring**: Regular check-ups with your healthcare provider can help detect and manage conditions that may predispose you to frozen shoulder.
- **Early Intervention**: Seek prompt medical attention if you notice early signs of shoulder pain or stiffness to prevent progression.

8. Stress Management

- **Relaxation Techniques**: Practice stress-relief techniques such as yoga, meditation, or deep breathing exercises to reduce muscle tension.

By incorporating these preventive measures into your daily routine, you can significantly reduce the risk of developing adhesive capsulitis. Maintaining shoulder mobility, addressing underlying health conditions, and staying active are key components in preventing this condition. If you are at high risk or have a history of shoulder issues, consulting with a healthcare professional for personalized advice and preventive strategies is recommended.

13. How often does frozen recur?

Frozen shoulder (adhesive capsulitis) is generally considered a self-limiting condition, meaning it typically resolves over time with appropriate treatment. However, the recurrence of frozen shoulder in the same shoulder is relatively rare. Here are some key points regarding the recurrence of frozen shoulder:

Recurrence in the Same Shoulder

- **Low Recurrence Rate**: Once frozen shoulder has resolved, it is uncommon for it to recur in the same shoulder. Studies suggest that the recurrence rate in the same shoulder is relatively low, estimated to be around 5-10%.

Bilateral Occurrence

- **Contralateral Shoulder**: It is more common for frozen shoulder to develop in the opposite shoulder at some point. This can occur in approximately 6-34% of patients who have had frozen shoulder in one shoulder. The time interval between the involvement of the two shoulders can vary widely.

Risk Factors for Recurrence

1. **Diabetes**: Individuals with diabetes are at a higher risk for both developing frozen shoulder and experiencing it in both shoulders.
2. **Systemic Diseases**: Conditions such as thyroid disorders and other systemic diseases may increase the likelihood of recurrence or bilateral involvement.
3. **Age and Gender**: Middle-aged individuals and women are more commonly affected by frozen shoulder, but these demographic factors do not necessarily influence recurrence rates.

Prevention of Recurrence

To minimize the risk of recurrence or bilateral involvement, consider the following measures:

1. **Maintain Shoulder Mobility**: Regular exercises to maintain flexibility and strength in the shoulders.
2. **Early Intervention**: Prompt treatment of any shoulder pain or stiffness to prevent progression to frozen shoulder.
3. **Manage Underlying Conditions**: Effective management of conditions like diabetes and thyroid disorders.
4. **Physical Therapy**: Continuing with physical therapy exercises even after recovery to maintain shoulder health.

Summary

While the recurrence of frozen shoulder in the same shoulder is uncommon, there is a higher likelihood of developing it in the opposite shoulder. Maintaining good shoulder health through regular exercise, early intervention, and management of underlying health conditions can help reduce the risk of recurrence and bilateral involvement. If you have had frozen shoulder before, discussing preventive strategies with your healthcare provider is advisable.

14. Under what circumstances should surgery be considered for treating frozen shoulder?

Surgery for treating frozen shoulder (adhesive capsulitis) is generally considered a last resort and is typically recommended only when non-surgical treatments have failed to provide significant relief over an extended period. Here are the specific circumstances under which surgery might be considered:

1. Prolonged Symptoms

- **Duration**: Surgery may be considered if symptoms persist for more than 6 to 12 months despite consistent non-surgical treatment, including physical therapy, medications, and injections.
- **Severity**: Severe and debilitating pain or significant loss of shoulder function that interferes with daily activities and quality of life.

2. Non-Responsive to Conservative Treatments

- **Ineffective Physical Therapy**: When extensive physical therapy fails to improve range of motion and reduce pain.

- **Inadequate Relief from Injections**: When corticosteroid injections, hydrodilatation, or other injections do not provide sufficient relief.

3. Severe Stiffness and Pain

- **Range of Motion**: Considerable limitation in shoulder movement that significantly restricts daily activities and does not improve with other treatments.
- **Pain**: Persistent and severe pain that does not respond to medications and other conservative measures.

4. Underlying Causes

- **Secondary Causes**: If the frozen shoulder is secondary to another condition such as a rotator cuff tear or other structural problems that might need to be addressed surgically.

Surgical Options

If surgery is deemed necessary, the following procedures might be considered:

1. **Manipulation Under Anesthesia (MUA)**
   - **Procedure**: The patient is placed under general anesthesia, and the surgeon forcefully moves the shoulder to break up adhesions and improve range of motion.
   - **Indications**: Typically used when there is severe stiffness and pain, and conservative treatments have not been effective.
2. **Arthroscopic Capsular Release**
   - **Procedure**: A minimally invasive surgery where a small camera (arthroscope) and surgical instruments are inserted through tiny incisions to cut through the tight or thickened parts of the shoulder capsule.
   - **Indications**: Considered when MUA is not appropriate or has failed to provide adequate results. It is often preferred due to its precision and lower risk of complications.

Post-Surgical Considerations

- **Rehabilitation**: Intensive physical therapy is essential after surgery to maintain the improvements in range of motion and prevent recurrence.
- **Recovery Time**: Recovery from surgery can vary, but most patients see significant improvement in pain and mobility within a few months. Full recovery can take several months, during which time regular follow-ups and adherence to rehabilitation protocols are crucial.

Summary

Surgery for frozen shoulder should be considered only when conservative treatments have failed to provide significant relief, and the condition severely impacts daily activities and quality of life. Consulting with an orthopedic surgeon who specializes in shoulder conditions can help determine the most appropriate treatment approach based on the individual’s specific circumstances and overall health.
